# Introducing Data Sciences to N-of-1 Designs, Statistics, Use-Cases, the Future, and the Moniker ‘N-of-1’ Trial

**DOI:** 10.1162/99608f92.116c43fe

**Published:** 2022-09-08

**Authors:** Karina W. Davidson, Ying Kuen Cheung, Ciarán P. Friel, Jerry Suls

**Affiliations:** 1 Institute of Health System Science, Feinstein Institutes for Medical Research, Northwell Health; Manhasset, NY; 2 Mailman School of Public Health, Columbia University; New York, NY

**Keywords:** between-subject designs, N-of-1 trials, personalized trials, single-patient trials, within-subject trials

## Abstract

This article, an introduction to *HDSR*’s “Personalized (N-of-1) Trials: Methods, Applications, and Impact” special issue, describes the rationale for a primer of the methods, data types and management, designs, and use cases for personalized (N-of-1) trials. It explains that the design and implementation of personalized (N-of-1) trials is only useful if patients volunteer for research involving them and actively participate in clinical services that use them. However, ‘N-of-1 trials’ may be an inadequate name to enact such patient engagement. The authors briefly review what patients have reported about the ‘N-of-1’ label and propose a more consumer-friendly moniker for this type of research and clinical approach to improve evidence-based science.

## 1. The Big Picture: From Clinical Encounter to Data Science

Successful therapy for a number of highly prevalent and chronic symptoms, risk factors, and health conditions has eluded us thus far. We postulate that the introduction of systematic data collection to assess the success of potential therapies—obtained from a single-patient or personalized trial—will increase the successful management of these health conditions, improve therapeutic precision, avoid use of unhelpful medications, and minimize individual patient harm. The clinical encounter, cherished though it is, has changed little in the many centuries that clinicians have provided care to patients. Whether physiologic or patient-reported, recent advances in data science over the past few decades can radically alter the clinical encounter by updating the available data on which to base therapeutic decisions for both clinicians and patients. In the future, we imagine a curated database combining data from similar personalized trials, which scientists can utilize to generate new knowledge, and clinicians can search on pertinent dimensions to review the results of revious personalized trials conducted with patients similar to theirs. The development of a single-patient, personalized, methodological approach that is automated and can be used at point-of-care could revolutionize the way we interact with patients and set the stage for a truly transformative approach to precision therapeutics. We also envision a future in which patients could avail themselves of these tools to conduct their own personalized experiments to test hypotheses about which therapies, whether behavioral or involving over-the-counter pharmaceuticals, provide them with the best results.

## 2. From Subjective to Systematic Data Collection in the Clinical Encounter

Providing data to clinicians, patients, and scientists is not radical; building a system powerful and flexible enough to allow many treatments to be tested, and that can be ordered with results returned remotely such that therapy decisions need not be made in a clinician’s office, is exceptionally radical. Additionally, this new paradigm becomes an opportunity for systematic, rather than subjective, data collection. Although the shift will face many cultural and logistical barriers, the time has come to introduce the scientific method to the clinical encounter when the efficacy of a therapy is in question.

## 3. From Between-Subject to Within-Subject Designs

Most clinical research—and particularly research concerned with drug development and evidence-based medicine—has been collected with between-subject designs, which is the proper action when the universality assumption holds (i.e., that all humans respond similarly to a therapy). Said assumption, then, leads to the conclusion that the hypothesized mechanism, expected targeted effect, or theory from which the therapy is derived will predict the response in a specific patient. No one holds this assumption, but currently we have no scientific platform on which to collect the individually precise therapeutic response from a specific patient and then, with sufficient data from several patients, examine these data to test whether the universality assumption holds. Universality may hold in some cases, but when it fails, a new scientific paradigm needs to be built upon the uniqueness assumption—that one patient’s therapeutic response is largely independent of the next patient’s response. The promise of within-subject trials, and specifically, personalized (N-of-1) trials—randomized crossover trials conducted in a single patient that are aided by an automated, user-friendly platform or system—offer an individualized, discovery-based phenotyping science to nurture a true instantiation of patient-centered care.

## 4. Why ‘N-of-1’ Trials Matter

Heterogeneous responses to the same treatment by individual patients, a plausible manifestation of the uniqueness assumption, are commonplace in biomedical research and patient care, and the phenomenon is quite familiar to statisticians, data scientists, and clinicians. The core of precision medicine is in many ways motivated by this fundamental problem, perhaps with a subtle shift in focus from a treatment-centered paradigm to a patient-centered paradigm. Rather than asking why individuals respond differently to the same treatment or need a different amount of the same treatment, a patient-centric approach asks what treatment works best for one particular individual. This approach is the impetus for examining the virtue of using personalized (‘N-of-1’) trials in this special issue. While the method to conduct N-of-1 trials appeared in the literature in the 1930s (Hogben & Sim, 1953; Martini, 1932; Mirza et al., 2017) and its practice has seen recent uptake, an update on the methods and statistical approaches of N-of-1 methodology is warranted; recent advances in analytical methods for data (e.g., Bayesian hierarchical modeling), data visualization (thanks to the advances in data science), and statistical designs (e.g., adaptive designs) can lend themselves to this increasingly important tool.

Two of the guest editors (Y.K.C. and K.W.D.) participated in an invited panel discussion at the 2018 Joint Statistical Meeting on “Small Data and N-of-1 Trials: Developing Personalized Biostatistics for Personalized Medicine and Individualized Health Care Delivery” (Schmid et al., 2018). The panel title itself is plain about the all-encompassing goal of advancing of medicine and public health. The solutions of the panelists, however, converged not on the leverage of big data but rather on a thoughtful experimental approach in the form of N-of-1 trials (or, as we prefer to refer to them, ‘personalized trials’). Many excellent and provocative use cases were presented and discussed at the panel, and they inspired thoughts about creating a standard text of methodologies for researchers to be able to carry out experimental studies properly and in a reproducible manner. Unsurprisingly, many of the panelists contributed to this special issue, as the idea to write such a special issue emerged during the workshop. While the field of within-subject—and, more specifically, personalized trials—is fast developing, this collection of articles will represent the state of the art and, more importantly, serve as the foundation of materials to be built upon.

## 5. What Personalized (‘N-of-1’) Trials Can Achieve in the Short Term

Personalized trials should be well suited to comparing interventions that, at the individual level, have the goal of improving health behaviors (e.g., physical activity and weight-reduction) that are otherwise expected to be relatively stable over time. Personalized trials can also be useful for determining whether to discontinue a treatment. In our era of polypharmacy—with many treatments offering unclear benefit—personalized trials designed to help dismantle a regimen may serve as a particularly promising use case.

A secondary benefit is that personalized trials can provide different treatment estimates than those derived from between-subject trial designs or conventional parallel-arm randomized controlled trials (RCTs). Evidence from conventional between-subject RCTs can conclude that the intervention tested was, on average, safe and effective, but the particular intervention may have had an uneven mix of risks and benefits to individual patients (Greenfield et al., 2007; Kravitz et al., 2004). This is indicative of an interaction between the treatment condition and individual participant characteristics. However, conventional RCTs generally provide limited power to detect such interactions, even in a meta-analysis combining the results from several studies (Altman & Bland, 2003). Understanding when heterogeneity of effects is occurring with medical and biobehavioral interventions will help researchers, clinicians, and policymakers identify those interventions or treatments that might best be studied by personalized trials rather than continuing to be tested with between-subject RCTs.

## 6. Thorny Issue With the Moniker ‘N-of-1’ Trial

“*I bet statisticians are going to still call it ‘N of 1,’ and that’s fine. They can do that in the lab. But for us patients, individual focus trial or something … sort of have a descriptive meaning … a* layman’s description.”

—Anonymous participant.

Researchers prefer the term ‘N-of-1’ for the kind of single-subject, within-subject crossover trial that this special issue highlights. Using a modified international Delphi approach, two standards—CONSORT (Consolidated Standards of Reporting Trials) and, more recently, SPIRIT (Standard Protocol Items: Recommendations for Interventional Trials)—have settled on this nomenclature to facilitate consistency in reporting N-of-1 trials. Clinical psychology has tended to use the term, ‘Single Case Experimental Design,’ for this type of trial; more recent consensus-based documents confirm the need for consistency in nomenclature between disciplines and have adopted/endorsed the use of N-of-1 trials for single-subject, within-subject multiple crossover trials (Tate, 2016). However, despite the apparent consensus of professionals in some quarters, our experience is that many laypeople, patients, physicians, and even scientists are often confused by the term. Furthermore, we fear that this confusion has dissuaded their use.

To understand how patients might view ‘N-of-1 trials,’ we conducted focus groups with patients and, separately, with primary care providers (Kronish et al., 2017). Our rationale for focus groups was that this methodology allowed us to use an interactive discussion to solicit key information about thoughts, attitudes, explanations, and beliefs. Further, it provided us with an opportunity to ‘meet people where they were’ on a complex and multifaceted subject for which elaboration and representation of the wide range of opinions on pertinent issues is essential (Clinical and Translational Science Awards Consortium, Community Engagement Key Function Committee Task Force on the Principles of Community Engagement, 2011; Powell & Single, 1996; Webb & Kevern, 2001). We did not require participants to have had prior experience engaging in N-of-1 trials, as we wished to understand the acceptability of this design and its name for those being asked to participate for the first time. Participants were first provided with a description of N-of-1 trials and then asked about their familiarity with N-of-1 trials, willingness to participate, perceived benefits and concerns with the N-of-1 design, and facilitators of involvement. Focus groups concluded by prompting participants to describe their suggestions for describing N-of-1 trials.

We asked 54 patients (24 English-speaking, 30 Spanish-speaking) in a series of focus groups about ‘N of 1’ trials. The majority were women, 43% had less than a college diploma, and 48% had an annual income of less than $60,000 year, indicating significant socioeconomic diversity (Table 1). We also enrolled 24 primary care providers (23 internists, 1 nurse practitioner). Their mean (standard deviation [*SD*]) age was 37.3 (8.6) years (range of 31 to 60 years), 61% were women, 4% were Hispanic, and 48% were White.

Few patients were familiar with N-of-1 trials and none had previously participated in one. Initially, some patients confused N-of-1 trials with more conventional RCTs. Despite this lack of familiarity, patients demonstrated an appropriate understanding of N-of-1 trials after being provided with a short description (see Appendix). While some clinicians were familiar with the concept of N-of-1 trials, the majority had not heard the term ‘N-of-1’ and none had conducted N-of-1 trials in their clinical practice. Nevertheless, clinicians quickly understood how N-of-1 trials represented a more rigorous approach to treatment selection than the trial-of-therapy approach that underlies usual clinical care.

Through our focus groups, we learned that patients and clinicians readily understood the potential benefits and use cases for N-of-1 trials, suggesting that there might yet be an opportunity to implement N-of-1 trials in clinical practice. Benefits that were highlighted included the potential to individualize treatment selection; increase patient engagement in self-care; foster better patient–doctor communication; and, ultimately, improve patient outcomes. Promoting N-of-1 trials as an approach to individualize care was highlighted by patients and clinicians as important to increasing acceptability. While patients and clinicians were enthusiastic about incorporating N-of-1 trials into clinical practice, they also identified concerns—most notably, about the time and cost burden of participation. Proponents of N-of-1 trials are well advised to heed these concerns in the materials they use to design and explain N-of-1 trials.

Patients and clinicians in our focus groups were unanimous in one theme only: they all opposed the name ‘N-of-1 trial’ to describe this design and approach to empirically determining a precise treatment effect. When asked to describe why they dislike the label, patients described that it sounded like they were going to be experimented upon, that it was an exploitative way of conducting science, and that it objectified them (i.e., by making them feel as though they were being treated like ‘guinea pigs’). Both patients and clinicians felt that the name failed to describe what the design accomplished. While clinicians had less active hatred of the name than did patients, they were not supportive of it. We then asked focus group participants to suggest better monikers. Suggested names included ‘individualized trial’; ‘signature trial’; ‘individual focus trial’; ‘single focus trial’; ‘patient-controlled trial’; ‘patient-doctor controlled trial’; ‘custom trial’; and ‘personalized trial,’ which was the most well liked.

A national patient survey was then conducted to determine the conditions, symptoms, and design attributes most applicable to N-of-1 trials according to patients (Derby et al., 2021). A sample of U.S. patients with two or more chronic medical conditions completed the online survey. On average, participants (*N* = 501) were 56.1 years of age and 57.2% were women, 7.6% were Black, 4.4% were Hispanic/Latino, 18.4% had a high school education or less, 43.0% were Medicare insured, and 18.8% were Medicaid insured. They responded about the conditions, symptoms, or use cases for N-of-1 trials. Participants were also asked to help identify a more intuitive and appealing name for the trial; they selected those they felt were best and worst out of the given options.

After reading a description of N-of-1 trials, 82.0% of participants were willing to participate in one. Such findings suggest that this trial design may help spur enrollment and retention of participants in federally funded clinical research, which has been an issue cited by the National Institutes of Health and others as critical to the equitable advancement of science and treatments.

Patient-selected names for these types of trials included ‘patient-/clinician-controlled trial’ (50.2%), ‘personalized trial’ (37.6%), and ‘individual focus trial’ (31.8%). Other potential names included ‘individualized trial’ (28.9%), ‘single patient–centered trial’ (22.9%), and ‘custom trial’ (18.7%). As clinicians, statisticians, and patients from the focus groups and the leadership team strongly preferred ‘personalized trials’ over ‘patient-clinician-controlled trials,’ we settled on the former to use as the name in the future. Thus, those proposing N-of-1 trials should be cognizant of the lack of appeal for ‘N-of-1 trials’ and may consider instead to dub them ‘personalized trials.’ This was not a set of studies conducted internationally, so the aversion to ‘N-of-1 trial’ cannot yet be generalized to all patients across the world.

By now, we hope readers wonder why this description about aversion to the name ‘N-of-1 trial’ and preference for the alternative ‘personalized trial’ is in an introduction to a special issue on such trials. Our approach was deliberate—from this point forward, we propose to ‘rebrand’ N-of-1 trials in discussions intended for lay audiences, as our research suggests it may be an unfitting name by which to describe these trials. The continued use of ‘N-of-1 trial’ may jeopardize its potential to improve our science, data accruement, and clinical management of patients. Therefore, we have asked our contributing authors to use ‘personalized trials’ as much as possible instead of ‘N-of-1 trials’ throughout the special issue. We will work with PubMed to determine if this term can be added to Medical Subject Headings (MeSH) terms. We will train future generations of data scientists, statisticians, and, importantly, patients to understand the benefits and limitations of personalized trials, and we will leave the name ‘N-of-1’ behind, particularly when addressing our primary target audience—patients.

## 7. Introducing the Special Issue on Advances in Personalized Trial Methods, Statistical Approaches, Data Science Involvement, and Pragmatic Conduct and Dissemination

In this issue, a syllabus is offered for teaching or training data scientists, methodologists, clinicians, and students about personalized trials. Single-case experimental design—and, more specifically, personalized trial methods, design considerations, and promise for improving health care—are considered in the first three articles (Epstein & Dallery, 2022; Cheung & Mitsumoto, 2022; and Duan et al., 2022). Data analytic and statistical approaches are covered in the next four articles (Schork, 2022; Schmid & Yang, 2022; Chandereng, 2022; and Moeyaert & Fingerhut, 2022). Best practices for reporting, ethics, and conduct of personalized trials comprise the next section (Porcino & Vohra, 2022; Samuel & Wootton, 2022; and Kravitz & Duan, 2022). Importantly, this issue concludes with use cases of personalized trials that range from presentation of results to patients, medication side-effect testing, lower back pain management, and multimorbidity disease management (D’Angelo et al., 2022; Howard et al., 2022; Butler et al., 2022; and Suls et al., 2022).

**Figure 1.**
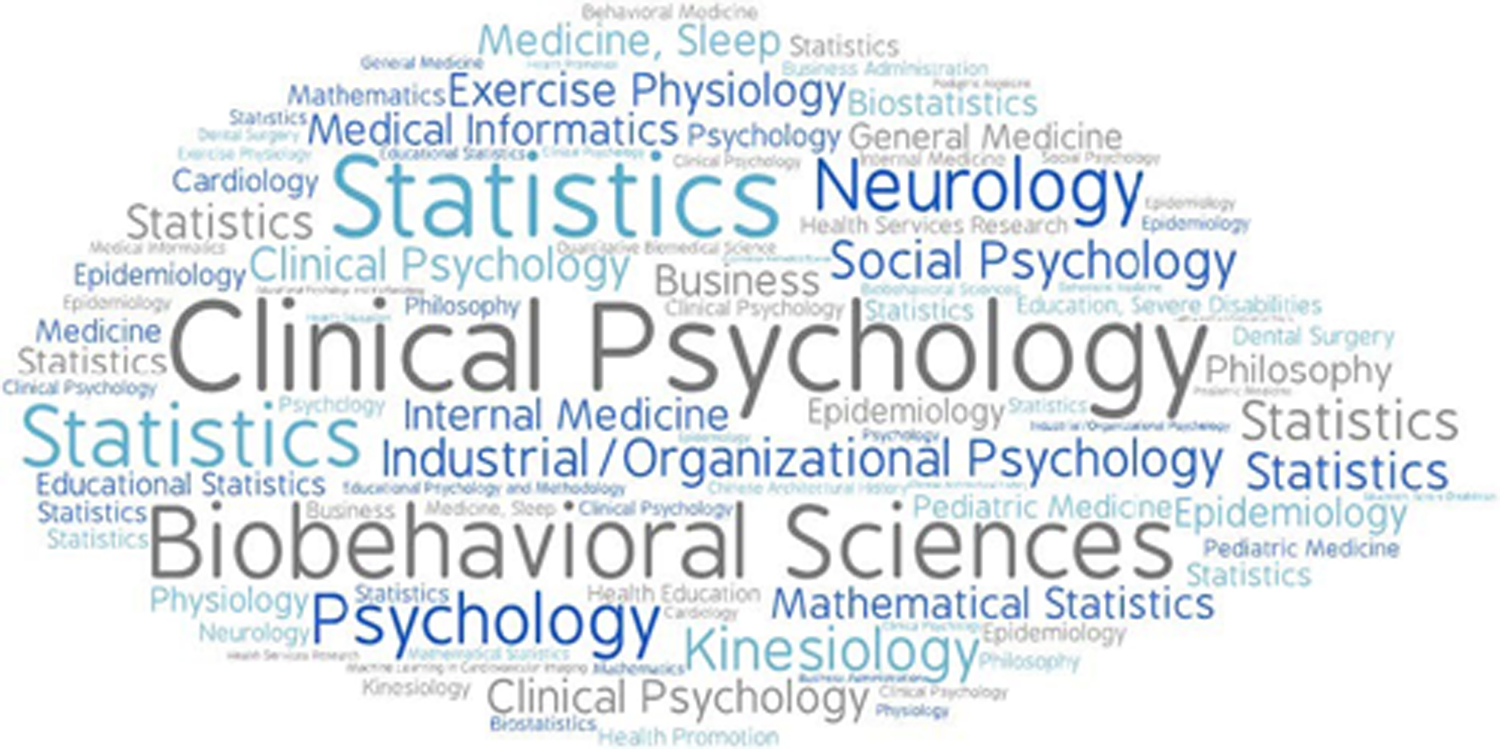
Word cloud of disciplines represented by contributing authors.

To illustrate the wide range of fields to which personalized trial methods can apply, we created a word cloud for the disciplines represented by the contributing authors (see Figure 1). The reader will note the inclusion of fields such as statistics, epidemiology, clinical psychology, internal medicine, medical informatics, public policy, philosophy, machine learning, and medical specialties in pediatrics, sleep medicine, and cardiac imaging.

Together, these articles provide a rich tapestry of advances that exist or are waiting to be tested and implemented by data scientists, clinicians, and their trainees. They collectively serve as a primer that should allow any inquisitive person with a roadmap to embark on a unique science that envisions a universe generalized to the single person rather than those not yet included in a sample. With the power of these new techniques, we are prepared to experimentally test for the optimal treatment with the most benefit and fewest side effects for a person needing treatment. We can, in many cases, abandon generalizing from past participants to the unique patient in immediate need. With this meta-assumption—that our statistical assumption is to the generalization of the future of one person rather than the generalization to all others—we can herald a new era of data science, intervention design, and clinical practice. We invite you to read this exciting (and, at times, controversial) foray into that future.

## Figures and Tables

**Table 1.** Demographic Information on Focus Group Participants

Characteristic	Primary Care Providers (N = 24)	English-Speaking Patients (N = 24)	Spanish-Speaking Patients (N = 30)
Age in years, mean (SD)	37.3 (8.6)	61.1 (11.7)	63.1 (14.8)
Female, %	60.9%	42.9%	60.0%
Hispanic, %	4.4%	0%	100%
White, %	47.8%	92.9%	92.0%
Education, %			
Less than college diploma	0%	42.9%	71.4%
College diploma or higher degree	100%	57.1%	28.6%
Income			
< $60,000/year	n/a	33.3%	55.9%
≥ $60,000/year	n/a	66.7%	44.1%

## Appendix Description Provided to Focus Groups and Survey Respondents

Most clinical trials are conducted by enrolling a large number of patients. Patients are assigned to one group, and each group receives one of the treatments being tested or, perhaps, no treatment at all. At the end of the study, the groups are compared in terms of how much each group improves on average. Additionally, after the study, participants often have the opportunity to have the treatment that was provided to the group who experienced the best results. In the newer design, each patient receives each of the treatments being tested in random order. Neither the doctor nor the patient knows the order of the treatments so as to ensure that any ideas either party already has about a treatment does not interfere with the results. As an example of this newer study type, consider a comparison of pain relief medicines to treat a patient with chronic pain. Said patient may receive Tylenol [week one], followed by Motrin [week two], followed by a placebo (or sugar in a pill) [week three], followed by another week of both Tylenol and Motrin. Each day throughout the study, the patient may have to answer several questions about pain to monitor how well each treatment is working. At the end of the trial, the treatment code is broken. Then, together, the patient and care provider can review the results, determine if the patient experiences better results with one treatment or another, and subsequently use the data to decide which would be best. Both the traditional and newer approach each have their pros and cons. Regardless of whether or not you have heard about or participated in these types of trials, we would like to ask you a couple of questions about your knowledge, attitudes, and beliefs about them.
